# Single-cell analyses uncover granularity of muscle stem cells

**DOI:** 10.12688/f1000research.20856.1

**Published:** 2020-01-21

**Authors:** John Saber, Alexander Y.T. Lin, Michael A. Rudnicki

**Affiliations:** 1 Sprott Centre for Stem Cell Research, Regenerative Medicine Program, Ottawa Hospital Research Institute, Ottawa, ON, K1H 8L6, Canada; 2 Department of Cellular and Molecular Medicine, Faculty of Medicine, University of Ottawa, Ottawa, ON, K1H 8M5, Canada

**Keywords:** satellite cells, heterogeneity, cell fate, single-cell analysis

## Abstract

Satellite cells are the main muscle-resident cells responsible for muscle regeneration. Much research has described this population as being heterogeneous, but little is known about the different roles each subpopulation plays. Recent advances in the field have utilized the power of single-cell analysis to better describe and functionally characterize subpopulations of satellite cells as well as other cell groups comprising the muscle tissue. Furthermore, emerging technologies are opening the door to answering as-yet-unresolved questions pertaining to satellite cell heterogeneity and cell fate decisions.

## Introduction

Over the past decade, developmental biology has furthered our understanding of the origins of satellite cells (or muscle stem cells) and the mechanisms that govern their quiescence, activation, and differentiation ^1^. In adult homeostasis, satellite cells are quiescent and express the nodal transcription factor Pax7 ^2– 4^. Upon physiological insult, such as an injury or exercise, satellite cells become activated, enter the cell cycle, and generate myogenic progenitors. The activation of satellite cells has long been believed to be linear, with a sequential cascade of transcription factor expression ^5^. Activated satellite cells become committed progenitors, also known as myoblasts, that express Myf5 and MyoD as well as Myogenin upon entering the differentiation program to become post-mitotic myocytes ^6^. Myocytes will fuse to form myotubes that ultimately create the scaffold for muscle tissue ^7^. Moreover, satellite cells are able to self-renew, allowing the long-term maintenance of the stem cell pool. Outside of these intrinsic molecules that determine satellite cell states, extrinsic cues also influence division kinetics and satellite cell commitment and differentiation ^8– 10^. Both intrinsic and extrinsic factors are crucial for satellite cell function, and both are impacted in disease backgrounds, such as Duchenne’s muscular dystrophy and aging ^11^. In this review, we will discuss recent insights into the molecular control of satellite cell function and how the emergence of single-cell technologies is impacting the discovery of novel cellular functions and cell fates.

## Known heterogeneity in the satellite cell population

**Figure 1.**
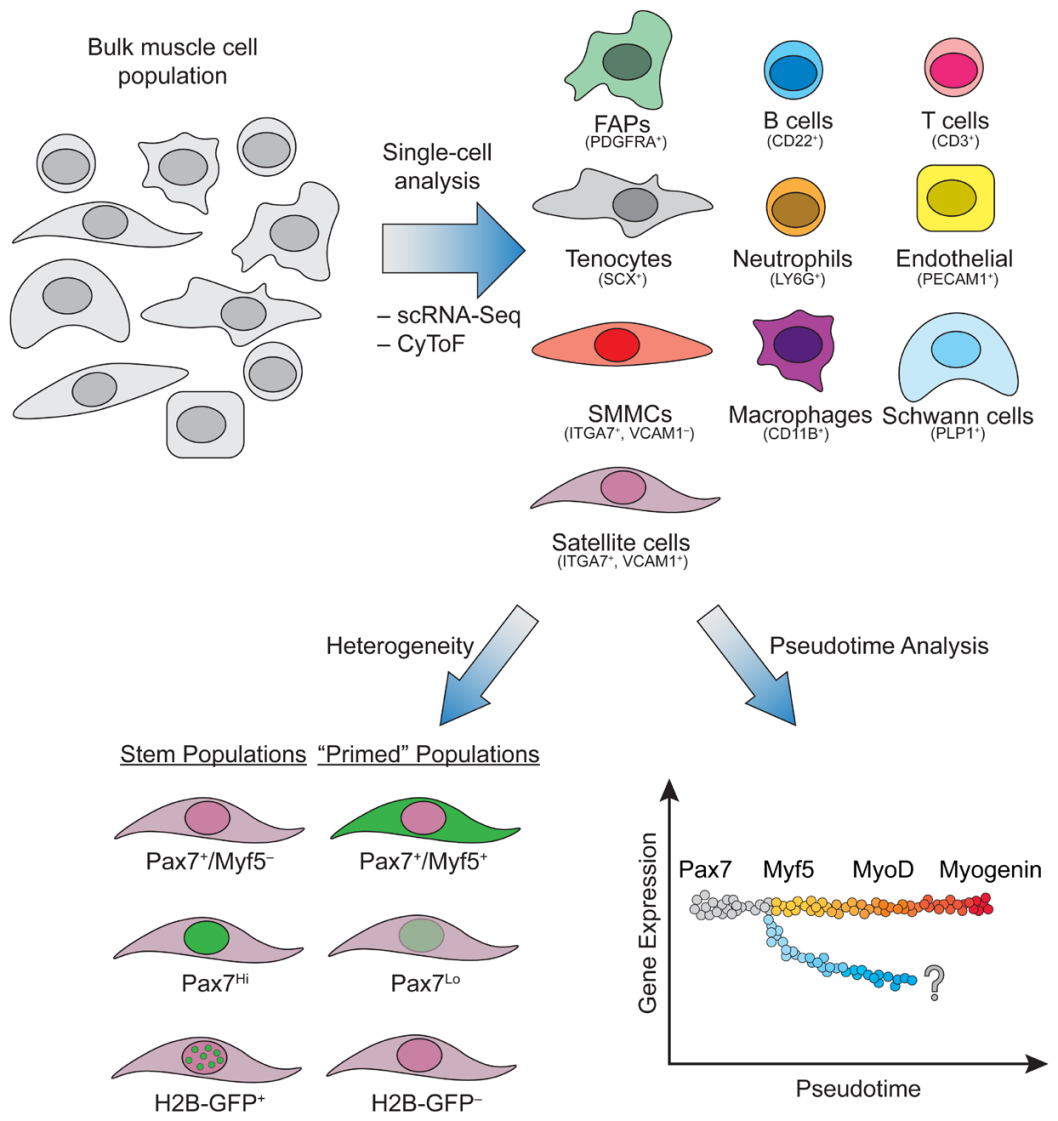
Single-cell analysis allows the identification of cell types residing in skeletal muscle. Top: Tools such as single-cell RNA sequencing (scRNA-Seq) and cytometry time of flight (CyToF) have been used to determine the identity of numerous cell types residing in skeletal muscle based on their transcriptional and protein signature. Notably, in addition to muscle fibers, muscle comprises satellite cells, immune cells, fibroblasts, endothelial cells, and Schwann cells. Bottom: The heterogeneity within the satellite cell population can be analyzed based on known markers associated with the stem cell state (Pax7 ^+^/Myf5 ^–^, Pax7 ^Hi^, and H2B-GFP ^+^ label-retaining cells). Furthermore, different cell states for satellite cells can be inferred using pseudotime analysis of existing transcriptional and proteomic datasets. Cells along the gray-to-red gradient represent the known cascade of transcription factor expression following the differentiation of satellite cells into myocytes. The diverging blue gradient illustrates potential unknown cell fates. FAP, fibro-adipogenic progenitor; SMMC, smooth muscle and mesenchymal cell.

Satellite cells are the main powerhouse for skeletal muscle regeneration. How satellite cells achieve this remarkable regenerative ability of generating progenitors yet maintain and balance self-renewal capacity can be posited as either stochastic fate acquisition or a hierarchical organization of asymmetric divisions with determined cell fates ^12^. Over the past decade, studies have demonstrated that satellite cells are organized hierarchically, whereby heterogeneity exists within this seemingly homogeneous Pax7 ^+^ population with functional subpopulations. Firstly, satellite cells have bi-potential activity and can generate both brown fat and muscle, which alludes to multiple differentiation trajectories ^4, 13^. In addition, subsets of Pax7-expressing satellite cells have differing functional potential ( Figure 1, Bottom). Two such subpopulations are 1) Pax7 ^+^/Myf5 ^–^ cells and 2) a Pax7 ^Hi^ group observed upon taking the top ~10% of GFP ^+^ cells from Pax7-nGFP mice, both of which show greater stemness upon transplantation, as determined by their ability to repopulate the stem cell pool ^14, 15^. It remains unknown whether other functional subpopulations that differ in self-renewal capacity exist in the total Pax7 ^+^ population. Furthermore, some satellite cells have long-term label retention based on H2B-GFP ^16^, while another rare subpopulation is Pax3 ^+^ and is resistant to radiation and to genotoxic stress ^17, 18^. All these findings suggest a functional hierarchical classification of satellite cells, which was reaffirmed by using the Pax7-CreER;R26RBrainbow2.1 mouse, where clonal dominance arose after repetitive injuries but clonal diversity was retained in homeostasis ^19^. However, it has been difficult to assess the functional subpopulations of satellite cells and their relationship to one another. The application of single-cell technologies should clarify these conundrums.

## Recent advances in muscle regeneration using single-cell technologies

The question of how complex tissues are formed from the basic individual units of cells has been advanced with the recent explosion in single-cell biology. Tissues are heterogeneous in composition, and studying cells at single-cell resolution facilitates insights into understanding individual cellular functions and their lineage relationships. These two concepts of function and lineage relationships have propelled many recent studies to discover new cell types and states, refined differentiation hierarchies during development and regeneration, and identified populations that are aberrant in disease contexts. Here, we will primarily focus on the two most mature and commercially available single-cell technologies: single-cell RNA-sequencing (scRNA-Seq) and single-cell proteomics with cytometry by time of flight (CyTOF). Both of these two technologies enable researchers to stratify heterogeneous populations within the total tissue and extrapolate trajectories with pseudotime algorithms to infer dynamic changes from static snapshots of specific populations.

## Current application of single-cell technologies on satellite cells

The recent application of single-cell technologies to study satellite cells, and by association and more broadly speaking muscle tissue, makes it possible to functionally delineate hierarchies and identify molecular programs governing sequential cell fates. Though studying single satellite cells is not a new idea, previous experimental designs and methods were not high throughput and limited the possibility of highly quantitative results. The first findings pertaining to single-cell analysis of satellite cells captured single cells from a muscle fiber using a micropipette, which is an extremely tedious and low-throughput endeavor ^20^. A contemporary paper isolated Tomato ^+^ cells from Pax7-CreER;R26RLSL-tdTomato mice and captured them on the Fluidigm C1 platform ^21^. However, their platform captured only 21 cells which did not cluster the population into any meaningful subpopulations. As the authors suggested, and where more recent studies have explored, the use of higher-throughput capture methods, primarily using droplet-based systems to capture single cells including Dropseq or 10X Genomics platforms, has been invaluable in identifying these subpopulations.

Two large-scale studies profiled a multitude of adult organs and tissues using a microwell-based method or droplet-based scRNA-Seq. Each group identified a cluster of muscle cells; however, neither study presented novel stratification or discovery of the muscle stem cell populations ^22, 23^. Using the Tabula Muris Consortium dataset, one group was able to validate a Twist2 ^+^ population that expressed Nrp1 to allow for selective fusion and generation of type IIB fibers ^22, 24, 25^. The first thorough study captured and performed scRNA-Seq on over 12,000 mononuclear cells from adult hindlimb muscle with complementary profiling using CyTOF ^26^. The combination of an X-shift clustering algorithm based on CyTOF data and scRNA-Seq clustering identified 10 major populations: B and T cells, macrophages, endothelial cells, fibro-adipogenic progenitors (FAPs), neutrophils, muscle stem (satellite) cells, integrin-α7 ^+^/VCAM1 ^–^ cells, Schwann cells, and an uncharacterized population negative for canonical markers ( Figure 1, Top). Interestingly, these undiscovered integrin-α7 ^+^/VCAM1 ^–^ cells display myogenic potential *in vitro*, though they are auxiliary to satellite cells during regeneration, as they enhanced satellite cell transplantation potential but failed to transplant as an isolated population. These integrin-α7 ^+^/VCAM1 ^–^ cells have a transcriptomic signature more akin to mesenchymal cells and have been aptly named smooth muscle and mesenchymal cells (SMMCs). This study also found an interstitial Scleraxis ^+^ population expressing tenocyte markers, which broadly falls under the fibroblast classification and may cooperate with FAPs to promote muscle regeneration. Probing resident muscle cells with two powerful single-cell technologies, CyTOF and scRNA-Seq, resulted in the identification of two uncharacterized populations.

The power of single-cell analyses can also refine differentiation trajectories and capture cell state transitions ( Figure 1, Bottom). A study from the Blau laboratory used CyTOF as a discovery tool and conducted a screen for new cell surface markers expressed in satellite cells and cultured myoblasts ^27^. Using the classical myogenic transcription factors—Pax7, Myf5, Myod, and Myogenin—to infer their clustering and population trajectories, they identified two progenitor populations: P1 and P2. P1 is Pax7 ^Lo^, Myf5 ^Hi^, MyoD ^Lo^, and Myogenin ^Hi^ and is marked by CD9, while the P2 population is Pax7 ^Lo^, Myf5 ^Hi^, MyoD ^Hi^, and Myogenin ^Hi^ and is distinguished by the presence of both CD9 and CD104 (or integrin beta 4). Interestingly, the P1 population seems to appear before the P2 population, despite seemingly turning off the expression of MyoD. The presence of Myogenin and absence of MyoD would suggest that the P1 population is further along the differentiation pathway, contrary to the proposed dynamics. However, both populations seem to exist at later stages of the myogenic lineage, since they both lack IdU incorporation following injury, confirming they do not enter the cell cycle and may both represent variations of post-mitotic myocytes.

**Figure 2.**
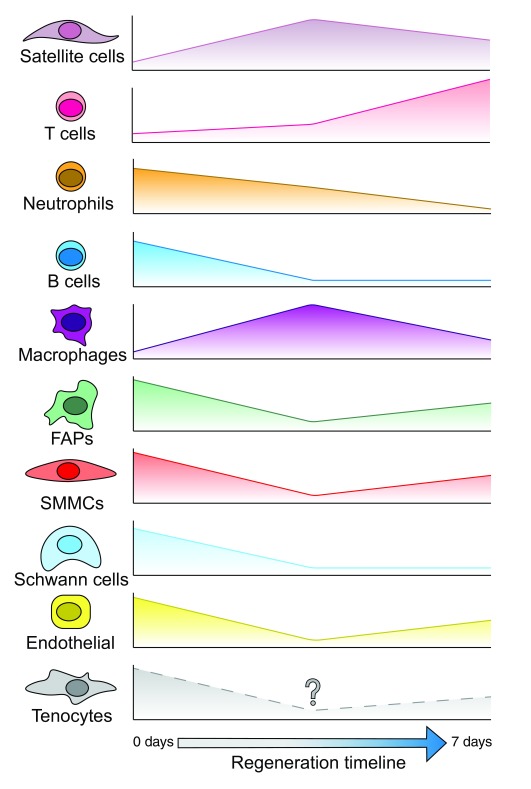
Dynamics of muscle-resident cells during regeneration. Single-cell RNA sequencing has been used to describe the dynamics of multiple muscle-resident cells through regeneration following injury ^28, 29^. Plotted are the relative proportion of each cell type compared to the bulk cell population at each stage. Some cells, such as satellite cells and T cells, make up a higher proportion of the population following injury. Others, like B cells and fibro-adipogenic progenitors (FAPs), seem to be comparatively reduced in numbers. It is unknown how tenocytes react during muscle regeneration. However, they may follow the same trend as FAPs owing to them not being discriminated from FAPs in the fibroblast compartment.

Complementary approaches to the experiments described above have been conducted to infer activation and differentiation hierarchies with scRNA-Seq. A few recent studies started by profiling the total muscle over a regeneration time course to assess populations that temporally change ^28, 29^. Both studies saw cell-type-specific clusters emerge and disappear throughout the progress of regeneration, reinforcing the idea that muscle regeneration is a dynamic process ( Figure 2). However, these results seem to contrast with previous publications regarding cell dynamics, possibly owing to the different injury models or to limitations in inferring cell types based solely on transcriptional profiling ^30^. For example, FAPs have been shown to increase proportionally to myogenic progenitors following an injury rather than decrease ^31^. However, this discrepancy might be due to scRNA-Seq being heavily skewed by other cells, whereby changes in cell dynamics are relative rather than absolute. Furthermore, they miss certain cell types such as tenocytes, possibly because they are grouped together with the FAP population as fibroblasts ^26^. Two groups then focused on satellite cell activation and used pseudotime algorithms to reconstruct differentiation trajectories. The study from the Sartorelli lab profiled homeostatic and 60-hour post-notexin-injured satellite cells and cultured myoblasts ^32^; their analyses reaffirmed that metabolic changes drive the activation of satellite cells ^33, 34^. However, one interesting finding was a split in their trajectory for cultured primary myoblasts, with one branch representing differentiation categorized by *Myogenin* expression, while the other branch retained *cyclinD1* and *D2*. The authors suggest that this latter branch represents the previously identified “reserve cells” that may represent a self-renewing population *in vitro* ^35^. It would be interesting to probe for the molecules enriched in these “reserve cells”, since they may elucidate pathways satellite cells undergo to return to quiescence once activated. Another study was able to parse the heterogeneity of the Syndecan family (1–4) and their differential expression among quiescent, activated, cycling, and committed satellite and their progenitor cells ^28^. Notably, the authors show that quiescent satellite cells can be divided into subpopulations that express Sdc2 and Sdc3 and those that don’t. Finally, another focused on an aging context and demonstrated that aged T-cells and satellite cells are transcriptomically more similar to their respective young and injured cell type, alluding to the possibility that these predispositions in aged contexts could be causative of poor regenerative outcomes ^29^. These descriptive studies using scRNA-Seq predominantly reiterate previous known molecules and paradigms in muscle regeneration ^36^.

More interestingly, one study combined scRNA-Seq with subsequent functional validation using single-cell time lapse imaging and provided insights contrary to convention ^37^. The authors focused on the differences between young and aged satellite cells and the transition from quiescence to activation. One noteworthy observation was that *Pax7* does not monotonically decrease upon activation, whereas quiescence-associated *Spry1* monotonically decreases. With the use of single-cell imaging and immunostaining, they found that the most motile and activated cells were actually enriched for Pax7. Thus, by conducting orthogonal studies on single cells, the authors drew on a strength and were able to associate specific molecular features with behavior. Another interesting finding was that both label-retaining cells (LRCs) and nonLRCs occupy the same transcriptional space, such that the two populations do not form distinct clusters based on their label retention function. Surprisingly, the activated cell cluster largely comprised LRCs, suggesting their increased ability to enter the cell cycle, which was reconfirmed with EdU experiments. How and why LRCs are able to activate quicker yet maintain label retention is an interesting and unresolved question. Finally, the study posits whether aged and young satellite cells have different trajectories and states or merely arrive at the same state albeit at different rates. Both aged and young samples overlapped in their trajectories, suggesting that cell state transitions were similar but the progression or rate of activation was slower in aged satellite cells.

## Emerging technologies

To fully describe satellite cells and other cells residing in muscle, as well as their overall function, current approaches based predominantly on scRNA-Seq are insufficient. Multimodal approaches, where multiple facets of the cell are considered simultaneously, will be needed to better understand the relationship among DNA structure, its impact on transcription, and the resulting proteins being formed that discriminate one cell from another. Currently, only the study by Giordani *et al.* has scratched the surface in muscle by profiling resident muscle cells using scRNA-Seq and CyToF ^26^.

However, other techniques currently being used in other fields can shed light onto the next steps of multimodal research in satellite cells and muscle regeneration. Single-cell analysis for RNA and protein (CITE-seq) has been described, allowing the simultaneous quantification of RNA transcripts and protein products in a single cell ^38^. This relies on the detection of oligonucleotide-labeled antibodies for the identification of proteins using similar workflow to scRNA-Seq. However, this technique allows the detection of cell surface proteins only, which limits its use for investigating differences in gene regulation. Another study by Genshaft *et al.* used proximity extension assays (PEAs, similar to proximity ligation assay) to evaluate intracellular protein levels by measuring the generation of a DNA reporter following the interaction of two antibodies targeting the same protein ^39^. This allows the simultaneous detection of proteins and RNA from single cells. However, this technique is limited to a small panel of proteins. Nevertheless, performing similar experiments in satellite cells can help identify some of the molecular differences between different subpopulations. For example, one can test the stemness of the Myf5 ^Lo^ or Pax7 ^Hi^ populations by simultaneously investigating the expression of many genes involved in cellular quiescence.

Chromatin accessibility at the single-cell level can also complement scRNA-Seq data in identifying regulators of cell fate. In addition to being present at transcription start sites, it is known that chromatin accessibility determined by DNase hypersensitivity sites is also localized to distal regions, suggesting a regulatory role in gene transcription rather than simply a direct effect on gene transcription ^40^. Thus, obtaining relevant single-cell accessibility information is relevant for deconvoluting the epigenetic mechanisms governing gene transcription in satellite cells, whether it be for understanding heterogeneity or determining modulators of cell fate. So far, no such experiments have been conducted in muscle, but other areas of research have put such techniques to the test. Single-cell assay for transposase-accessible chromatin using sequencing (ATAC-Seq) coupled with scRNA-Seq have allowed the identification of gene expression and chromatin accessibility from the same cell ^41^.

Moreover, single-cell chromatin immunoprecipitation coupled with sequencing (scChIP-Seq) will be invaluable to complete the picture. One group has used scChIP-Seq to compare H3K27me3 patterns in cells originating from breast cancer tumors ^42^. Briefly, they found that a subset of cells from untreated tumors had a decrease in H3K27me3 levels, a pattern similar to cells from tumors that have developed drug resistance. This led to an increase in the expression of genes that are normally repressed. This study is a good example of the potential of scChIP-Seq in identifying cell heterogeneity. However, these current methods do not allow the simultaneous measurement of enough variables to obtain a full understanding from within the same cell. Therefore, future work bringing together scRNA-Seq, ChIP-Seq, and ATAC-Seq would be invaluable in painting a complete picture of the epigenetic landscape and its functional consequence on satellite cell gene expression.

Additionally, new imaging techniques are quickly gaining popularity for the investigation of single-cell function. Spatial-omics techniques are now able to capture gene expression at the single-cell level in relation to spatial information (MERFISH and Seurat) ^43, 44^. MERFISH and Seurat allow the integration of RNA-FISH data with scRNA-Seq, allowing the quantification of RNA with subcellular localization.

Lastly, many omics techniques rely on the isolation of cells of interest from the niche prior to data acquisitions. However, given the importance of the niche in the function of many cells, it is important to investigate the function of satellite cells while maintaining the niche. Few techniques allow this. One approach gaining popularity is the use of antibodies conjugated to oligonucleotides. This allows the specific detection of proteins while permitting multiplexing that would be impossible to achieve using conventional microscopy techniques. The first technique of its kind is CODEX, which uses tagged antibodies with double-stranded DNA containing specific overhangs ^45^. By carefully selecting the nucleotide sequence, this technique allows the iterative detection of antibodies—and thus proteins—based on the incorporation of a fluorophore-tagged nucleotide. Using this technique, the authors described a striking impact that the niche has on immune cell receptor expression from the spleen. An evolution of this technique is Immuno-SABER, whereby fluorophore-tagged imagers bind in a controlled manner to the target DNA sequence conjugated to an antibody ^46^. This technique has the advantage of not requiring *in situ* amplification of DNA and would also allow, in theory, the simultaneous detection of multiple proteins. Imaging mass cytometry is also a powerful tool to allow the acquisition of images of cells from bulk tissues without perturbation of the niche ^47^. Imaging mass cytometry has already been used to describe cancer cell subpopulations and cell–cell interactions, information which would be lost using sorting strategies ^47^. Coupled with the recently described muscle-resident cells, this would allow the imaging of dozens of markers per cell, and many different types of cells, to further elucidate the niche requirement in the function of satellite cells and better understand the dynamics of muscle regeneration ^26^. For example, do FAPs or macrophages modulate satellite cell function through direct interaction or paracrine signaling? Although current approaches do allow the investigation of such questions, they are incapable of multiplexing, an essential factor for considering the niche in its entirety given the complexity of cell types and the difficulty in inferring cell types solely based on gene transcription. Moreover, maintaining the niche is important for accurately studying satellite cells, since its disruption has a rapid, profound effect on their transcriptome ^48– 50^.

## Future perspectives

The current studies using single-cell technologies shed new light on novel muscle populations and prospective markers for their isolation but also reaffirm concepts and decades of functional regenerative myogenesis work without major paradigm-shifting outcomes. The discovery of these new cell types and states allows for further investigation into their ontogeny, such as CD9 ^+^ myogenic progenitors or SMMCs ^26, 27^. These single-cell studies also highlight that discovery-based tools are predominantly descriptive in nature and have thus far generated only tissue atlases. Trajectories and cell states determined by algorithms are only inferences and will require functional validation, suggesting that classical lineage tracing is not replaceable. Moreover, one of the pitfalls of transcriptomic trajectories is that true cellular lineages are mitotic cells and their descendants, such that scRNA-Seq may miss certain dynamisms. For instance, trajectory analysis is a forward pathway, yet self-renewing muscle stem cells must also move backwards—or loop—to return to quiescence, which currently has not been captured in these projections. Similarly, generating descendants from self-renewing mitotic stem cells at different times or accounting for proliferative history may also be lost in pseudotime analyses. Despite this, progress is being made in merging the inferred lineages using single-cell transcriptomics with single-cell genetic lineage tracing, potentially alleviating some of the issues with the inferences while still allowing high-throughput analysis ^51^.

Single-cell analyses still have a lot of utility in answering questions about muscle regeneration. For instance, the full story of satellite cell heterogeneity remains largely unclear. Functional differences have been documented ^14, 15, 17, 18^, yet only one study has been able to capture subpopulations of Pax7 ^Hi^ and Pax7 ^Lo^ cells ^32^. Other documented factors of heterogeneity have not been reflected in current single-cell approaches. Perhaps profiling large purified populations of satellite cells in combination with multiplexing or epitope tagging would provide more resolution. Accordingly, further investigation using CyTOF and markers associated with asymmetric cell divisions or intracellular signaling and phospho-proteins, such as Notch components or the PAR complexes, could further stratify homeostatic and injury-induced satellite cells. Other unresolved questions pertain to the regulation of multipotential specification, which has yet to be investigated using single-cell approaches, and characterizing cells in a disease state such as Duchenne’s muscular dystrophy. As previously mentioned, many new multimodal techniques also allow for simultaneous recordings of omics that can also be combined with spatial information. As these technologies keep maturing and become applicable and easily translatable to muscle stem cell biology, it will be an exciting time to keep refining the ideas in a context-dependent manner ranging from development to adult homeostasis, regeneration, and disease.

## Abbreviations

ATAC-Seq, assay for transposase-accessible chromatin using sequencing; CyTOF, cytometry time of flight; FAP, fibro-adipogenic progenitor; LRC, label-retaining cell; scChIP-Seq, single-cell chromatin immunoprecipitation coupled with sequencing; scRNA-Seq, single-cell RNA sequencing; SMMC, smooth muscle and mesenchymal cell.
